# MiR-98-5p/IGF2 Axis Influence Herceptin Sensitivity through IGF1R/HER2 Heterodimer Formation and AKT/mTOR Signal Pathway in HER2 Positive Breast Cancer

**DOI:** 10.31557/APJCP.2021.22.11.3693

**Published:** 2021-11

**Authors:** Mingliang Zhang, Zhixiang Li, Xianfu Liu

**Affiliations:** *Department of Oncology Surgery, First Affiliated Hospital of BengBu Medical College, BengBu city, AnHui province, China. *

**Keywords:** miR-98-5p/IGF2 axis, HER2 positive breast cancer, Herceptin resistance

## Abstract

**Background and Aim:**

IGF1R and HER2 are both members of the growth factor receptor family which is known to play a different role in breast cancer. However, correlation between IGF1R and HER2 has created a controversial situation that need to be fully delineated in development of Herceptin resistance. The aim of this study was to investigate the mechanism of Herceptin resistance through the IGF1R pathway in HER2 positive breast cancer.

**Materials and Methods:**

Clinical data were obtained from TCGA database and archived documents from The First Affiliated Hospital of Bengbu Medical College. Western blot and immunohistochemistry were used to detect proteins and their phosphorylation. Cell transfection were constructed using shRNA lentivirus vectors. RNAs were analyzed by RT-qPCR. Proteins in serum were analyzed by ELISA assay. Cell proliferation was analyzed by MTS method. Luciferase report experiment was conducted to verify RNA’s inter-reaction.

**Results:**

Western blot showed IGF2 protein was significantly increased in Herceptin resistant SKBR3-R cells (P<0.01), and IGF1R/HER2 heterodimer level was significantly increased (P<0.01). Luciferase reporter assay verified miR-98-5p could bind to 3 ‘UTR of IGF2 mRNA. When miR-98-5p was upregulated, the expression level of IGF2 was decreased(P<0.01), the cell invasive ability was reduced(P<0.01), and ultimately, Herceptin resistant cells regained their sensitivity to Herceptin. In clinical research, we found that decreased miR-98-5p level or increased IGF2 level significantly associated with poor treatment response and poor overall survival (OS), poor recurrence free survival (RFS) and poor distant metastasis-free survival (DMFS) in HER2-positive breast cancer. Conclusion: MiR-98-5p and IGF2 might potential candidates for predicting Herceptin sensitivity and provides a new way to overcome Herceptin resistance in clinic.

## Introduction

Breast cancer has overtaken lung cancer to become the most common malignancy, according to the latest statistics of global cancer incidence (Sung et al., 2021). It can be divided into different molecular sub-types and associates with different outcomes (Shim et al., 2014). Human epidermal growth factor receptor 2(HER2) positive breast cancer is characteristic of HER2 protein over-expression or gene amplification-account for 20-25% of newly diagnosed breast cancer approximately, and is significantly associated with poor prognosis ever before (Emi et al., 2002; Dawood et al., 2010). Herceptin as an effective anti-HER2 targeting agent has significantly improved survival of patients with HER2-positive breast cancer. Nowadays, HER2 has turned out to be somewhat of a good prognostic biomarker because of Herceptin treatment recommended by National Comprehensive Cancer Network (NCCN) breast cancer guideline (Gradishar et al., 2021). However, there is always a subset of patients with HER2-positive breast cancer who respond poorly to Herceptin, accounting for about 30-50% of total patients (Albrecht, 2010; Ahmad et al., 2014). This subset of patients who lose effective treatment eventually develop tumor recurrence and distant visceral metastases, which account for approximately 90% of breast cancer deaths (Ahmad et al., 2014). Therefore, Herceptin resistance has become a major obstacle to clinical treatment of HER2-positive breast cancer. Undoubtedly, searching for biomarkers that can effectively predict the sensitivity of Herceptin treatment and efficient ways to overcome drug resistance are making sense.

Insulin-like growth factor 2 (IGF2) is the endogenous ligand of insulin-like growth factor 1 (IGF1R). In our previous studies, we found that IGF1R/IRS1/AKT signal pathway was abnormally activated in Herceptin resistant cells (Zhang, 2017). It specifically binds to IGF-1R, triggering downstream mitogen-activated protein kinase (MAPK) and phosphoinosyl 3 kinase/protein kinase B(PI3K/Akt) signal transduction, thus regulating tumor cells growth, proliferation, invasion and metastasis (Sun et al., 2015; Spiliotaki et al., 2018). In recent study, researchers found that IGF2/IGF-1R/IRS1 signaling conferred Herceptin resistance in HER2-positive breast cancer (Luo et al., 2021). Although there are different points of view (Kostler et al., 2006). However, the factors influencing IGF2 expression level in tumor cells are not clear. MiR-98-5p is a microRNA that can regulated mRNA translation. Many studies have found that miR-98-5p can regulate the expression of intracellular proteins and inhibit tumor proliferation (Qiu et al., 2020; Huang et al., 2021; Zhan et al., 2021). In this study, we screened the target genes of miR-98-5p by Target Scan, and IGF2 mRNA was one of them. But relationship between them has not been reported before. Therefore, the purpose of this study is to reveal how miR-98-5p /IGF2 axis works and promotes the Herceptin resistance in cells. 

## Materials and Methods

*Cells and key reagents*

SKBR3 cell line was purchased from ATCC, USA, and stored in the conditional medium with DMEM containing 10% fetal bovine serum from Invitrogen, USA. Herceptin resistant cell line SKBR3-R derived from SKBR3 cultured with low concentration of Herceptin. RhIGF2 and antibodies were purchased from R&D Systems, USA and Cell Signaling Technology, USA. MiRNA mimics or inhibitors were purchased from Ribo-Bio, China. Lipofectamine 3000 was purchased from Invitrogen, USA. Western blot kit was purchased from Thermo Scientific, USA. All primers were designed with Primer3 Input and synthesized by Nanjing Kingsley Company. 

*Clinical analyses*

40 clinical samples were collected from the First Affiliated Hospital of Bengbu Medical College, admission time was from January 2019 to May 2021. All patients were initially diagnosed with HER2 positive breast cancer and used Herceptin-containing regimen as neoadjuvant treatment according to the guide line. Serum and tumor samples were collected under the full informed consent of the patients and the approval of the Human and Animal Ethics Committee of Bengbu Medical College (Approval number: [2020] No. 203). Patients were divided into two groups according to the response evaluation criteria in solid tumor (RECIST 1.1). The good response group consisted of patients with Complete Response (CR) and Partial Response (PR). The poor response group consisted of patients with Stable Disease (SD) and Progressive Disease (PD).

*Cell transfection*

We used lentivirus-mediated specific shRNA vectors to carry out cell transfection test and screened with puromycin (2μg/mL) to establish knockout or overexpressed transfection agents. MiRNA mimics or inhibitors were transfected into cells by Lipofectamine 3000 according to the instruction.

*Western blotting*

RIPA buffer was used to extract total proteins from cells. Protein concentration was measured by BCA Protein Assay Kit according to instruction. An equivalent amount of protein was added with 5×Lane Marker reduced sample buffer, then mixed with SDS-PAGE gel, and then transferred to PVDF membranes. The membranes were closed with 5% skimmed milk in Tris buffer brine and incubated with primary antibodies at 4^o^C overnight and secondary antibodies at normal temperature one hour. The signal protein detected by enhanced chemiluminescence Western Blot detection kit. The signal density was analyzed by ImageJ software (V2.0.0).

*Quantitative RT-PCR*

Total RNA and miRNA in cells were isolated by the RNA Isolation Kit (QIAGEN, Hilden, Germany) according to instructions. RT-QPCR was performed using the CFX96 Real-time PCR system (Foster City, California, USA), which using β-actin as internal control. The RNAs relative quantitative expression was measured and calculated by RNU6 normalization.

*Cell proliferation assay*

CellTiter 96^®^ AQueous One Solution Cell Proliferation Assay kit was purchased from Promega, USA, and we used it to analyze cell proliferation. According to the instructions in the kit, we seeded cells onto 96 well plates, each well was maintained with 2,000 cells and treated with different concentrations of Herceptin as part of the culture medium, then incubate with MTS for 2 days. The absorbance at 490nm was used as a reference value for cell viability, and calculated the cell viability at each preset point.

*Luciferase reporting assay*

The IGF2 3’UTR gene sequence was cloned into report plasmid located at downstream of firefly luciferase reporter gene. Cells were inoculated into 96-well plates, pMir-Report luciferase vector transfected into the cells. luciferase activity was measured after successful transfection was confirmed. using the dual luciferase reporter Assay system. We used the luciferase activity of Renilla as the internal control. 

*Co-immunoprecipitation*

Cells were lysed with modified TNE buffer containing interleukin (1 mg/L), aprotinin (1 mg/L), and Na3VO4(1 mM). Immunoprecipitation was performed at 4^o^C overnight with specific Ab or/and IgG as negative control. Immunoprecipitation was then incubated with protein G-agarose for about 2 h at normal temperature. We used a lysis buffer to wash the reaction products for 3 times, and the complexes of interacted proteins were then dissolved by SDS-PAGE, followed by Western blot analysis (see Western blot above).

*Immunohistochemistry*

The tissue was cut into 4μm sections after formalin fixation and paraffin embedding. they were separated and dewaxed in xylene and treated with different concentration of alcohols successively. Take off the slides and put into 62^o^C oven drying for 2h. After antigenic repair, 3% hydrogen peroxide was used to treat the slides in methanol for 15 minutes at normal temperature. The antigen was extracted according to the instruction. The film reading was completed by two senior pathologists independently. “0~+” consider to be low expression, “++~+++” consider to be high expression.

*Statistical analysis*

Data with normal distribution are shown as`x±s, and are calculated at least three times of independent experiments. Student’s t-test was used to compare difference between groups. Chi-square test was used to compare differences in expression rates. statistical package for social sciences (SPSS) version 19.0 and Excel 2019 were used for statistical analysis and graphics. p < 0.05 was considered statistically significant.

## Results

*IGF2 expression in HER2 positive breast cancer and correlation with prognosis*

**Figure 1:**
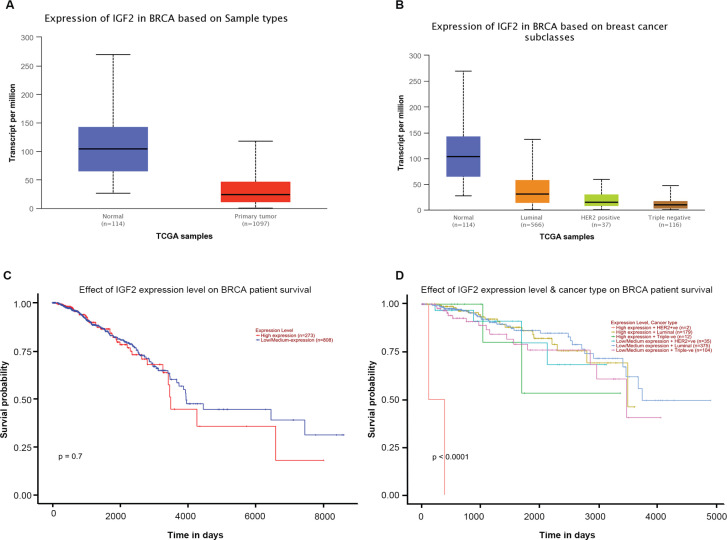
The Expression of IGF2 in Breast Cancer Tissues and the Relationship between Its Expression Level and Breast Cancer Survival, based on TCGA Database (n=1211). (A) IGF2 expression was lower in breast cancer than normal tissue. (B) IGF2 expression was lower in HER2 positive breast cancer than normal tissue. (C) The expression level of IGF2 was not related to the overall survival of breast. (D) The expression level of IGF2 was significantly correlated with the overall survival rate of HER2 positive breast cancer

We performed an analysis of IGF2 expression in breast cancer tissues through TCGA databases. The level of IGF2 expression was lower in breast cancer than normal tissue, regardless of any subtype (Figure 1A and B). There was no difference between IGF2 expression and overall survival of breast cancer (Figure 1C). But interestingly, patients with increased IGF2 expression had shorter survival times in the HER2-positive subgroup (Figure 1D). These results suggested that IGF2 expression might have important clinical significance specifically in HER2-positive breast cancer.

*IGF2 induces IGF-1R/IRS1/AKT signaling pathway activation through promotes IGF1R/HER2 heterodimer formation in SKBR3-R cells*

**Figure 2:**
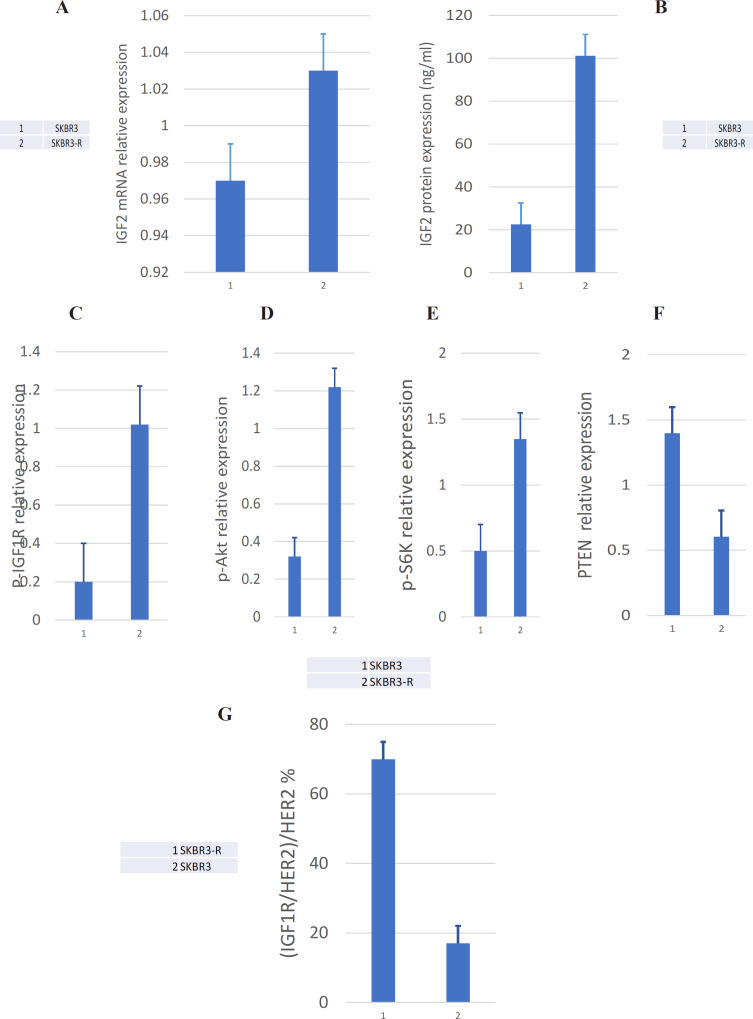
A, qRT-PCR assay showed that IGF1 mRNA expression level had no difference between SKBR3 and SKBR3-R cells. B, Western blot assay showed that IGF1 protein expression level in SKBR3-R cell was significantly higher than that in SKBR3 cell. C,D,E,F, Western blot assay showed that the phosphorylation level of p-IGF1R, p-IRS1, p-AKT, p-S6K in SKBR3-R cell were significantly higher than that in SKBR3 cell, while the expression of PTEN protein was significantly decreased. G, Co-Immunoprecipitation detection showed that the formation of IGF1R/HER2 heterodimer in SKBR3-R cell was significantly higher than that in SKBR3 cell

SKBR3-R cell resistant to Herceptin was reported in our previous study. To verify that SKBR3-R cells developed resistance to Herceptin, we treated the two strains with different concentrations of Herceptin. The IC_50_ values in SKBR3-R cell and SKBR3 cell were 220.53±12.21μg/ml and 23.52±2.02μg/ml, respectively (supplement Figure 1). The difference was significant (p<0.01). qRT-PCR assay showed the relative expression levels of IGF2 mRNA in SKBR3 and SKBR3-R cells were 0.97±0.44 and 1.03±0.38 respectively, with no significant difference (p>0.05) (Figure 2A). Interestingly, western blot assay showed the expression levels of IGF2 protein were 22.52±3.55 ng/mL and 101.12±12.02 ng/mL respectively in SKBR3 and SKBR3-R cells, and the difference was significant (p<0.01) (Figure 2B). This meant that IFG2 was regulated in protein translation. Further, we detected the protein phosphorylation expression of IGF1R downstream signaling pathway. Western blot showed that the expression of p-IGF1R in SKBR3 and SKBR3-R cells was 0.20±0.10 and 1.02±0.43 respectively (p<0.01), p-Akt(S473) was 0.32±0.12, 1.22±0.34 respectively (p<0.01), p-S6K was 0.50±0.21 and 1.35±0.60 respectively (p<0.01), PTEN was 1.39±0.55 and 0.60±0.15 respectively (p<0.05) (Figure 2C~F, supplement Figure 2). Elevated levels of phosphorylated proteins indicated activation of signaling pathways. To verify the interaction between IGF1R and HER2, we used Co-Immunoprecipitation detection which showed the formation of IGF1R/HER2 heterodimer in SKBR3-R cell was significantly higher than that in SKBR3 cell, the ratio of heterodimer to HER2 were (70±12.30) % and (17±1.95) % respectively, difference was significant (p<0.01) (Figure 2G, supplement Figure 3).

*MiR-98-5p regulated IGF2 expression and affected cell invasion and sensitivity to Herceptin*

**Figure 3:**
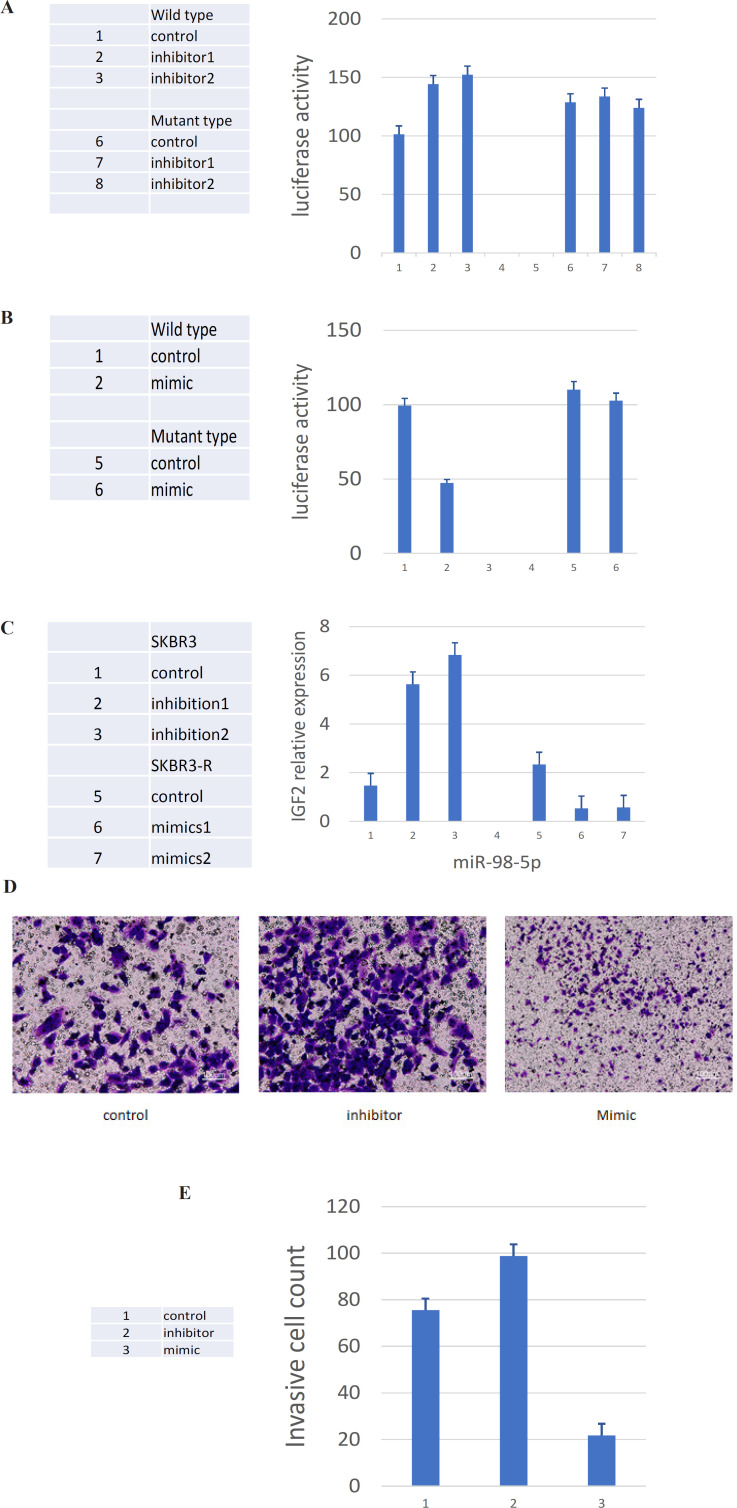
A, Luciferase report gene test showed luciferase activity was significantly increased in reporter gene containing wild type 3’UTR of IGF2 but not in the reporter gene with mutant binding site. B, Luciferase report gene test showed luciferase activity was significantly inhibited in reporter gene containing wild type 3’UTR of IGF2 but not in the reporter gene with mutant binding site. C, Western blot assay showed that IGF2 protein expression was significantly up-regulated by miR-98-5p inhibitors, and miR-98-5p mimics significantly down-regulated IGF2 protein expression.D, Cell invasion assay showed the number of cells invaded after transfusing miR-98-5p mimic and inhibitor (x 100). E,Cell invasion assay showed the number of cells invaded were significantly decreased after transfusing miR-98-5p mimic and increased after transfusing miR-98-5p inhibitor

We used “TargetScan” online database to explore specific MicroRNAs that target IGF2 (http://www.targetscan.org/vert_72/), the result showed miR-98-5p had a high possibility of targeted combination with 3’UTR sequence of IGF2 mRNA (supplement Figure 4). In order to determine whether miR-98-5p could directly target and regulate IGF2, we cloned the full length of 3’UTR sequence of IGF2 mRNA, which containing a wild or mutant miR-98-5p binding sequence, transfected into the firefly luciferase reporter plasmid. Firstly, we examined the effect of miR-98-5p inhibitors on luciferase activity in SKBR3 cells. The results showed that luciferase activity was significantly increased in reporter gene containing wild type 3’UTR of IGF2 but not in the reporter gene with mutant binding site (Figure 3A). Furthermore, we examined the effect of miR-98-5p mimics on luciferase activity in SKBR3 cells. The results showed that luciferase activity was significantly inhibited in reporter gene containing wild type 3’UTR of IGF2 but not in the reporter gene with mutant binding site (Figure 3B). These data suggested that miR-98-5p could regulate IGF2 expression through binding its 3’UTR region sequence. Further, lentiviral vectors were used to transfect miRNA mimics or inhibitors into cells, respectively. Firstly, we transfected miR-98-5p inhibitors into SKBR3 cells, the results showed IGF2 expression was significantly increased (p<0.05) (Figure 3C, supplement Figure 5), cell invasion assay showed the number of invaded cells were significantly increased (p<0.05) (Figure 3D-E), cell viability assay showed that cells were sensitive to Herceptin became resistant to the drug (supplement Figure 6). Secondly, we transfected miR-98-5p mimics into SKBR3-R cells, the results showed the IGF2 expression decreased significantly (p<0.05) (Figure 3C, supplement Figure 5), and the number of invaded cells were significantly decreased(p<0.05) (Figure 3D-E), cells’ resistance to Herceptin was reversed and they became sensitive to Herceptin (supplement Figure 7). These findings indicated that miR-98-5p might affect the HER2-positive breast cancer cell’s sensitivity to Herceptin and cellular malignant phenotype by regulating IGF2.

*Expression of IGF2 or miR-98-5p was significantly correlated with prognosis of HER2-positive breast cancer patients*

**Figure 4:**
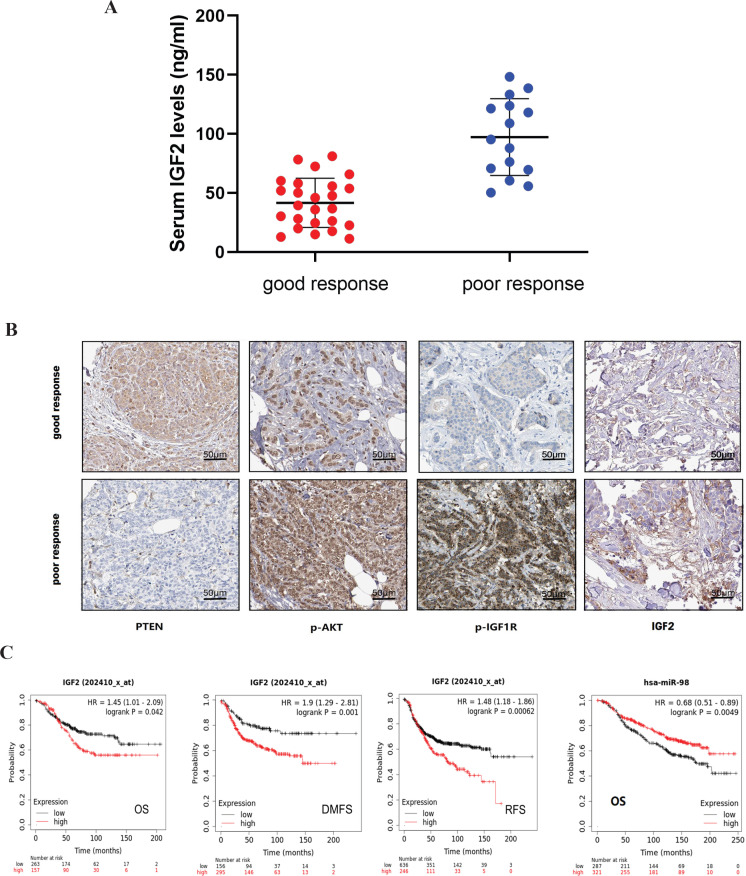
A, ELISA assay showed that serum IGF2 level in the good response group was lower than in the poor response group. B, Immunohistochemical staining showed that the level of IGF2, p-IGF1R, p-Akt(S473) decreased in good response group compared to in poor response group, but the expression of PTEN was just the opposite (x 50). C, Data from TCGA database showed IGF2 expression level was significantly correlated with the overall survival, distant metastasis-free survival, relapse-free survival, and miR-98-5p expression level was significantly correlated with overall survival specifically in patients with HER2-positive breast cancer

In order to explore the clinical importance of our results, we collected archived data of HER2-positive breast cancer in our hospital as the validation cohort (n=40, Tab 1) and online data from the TCGA-BRCA database (n=1,222, Tab 2). All the 40 patients were treated with neoadjuvant therapy containing Herceptin regimen and were divided into two groups as good response group (n=25) and poor response group (n=15) according to the RECIST 1.1 evaluation criteria (supplement Figure 8). ELISA assay showed that serum IGF2 levels were 41.63±20.82ng/ml in the good response group and 97.25±32.45 ng/ml in the poor response group, the difference was significant (p<0.01) (Figure 4A). Univariate analysis revealed that the expression of IGF2 was only related to therapeutic response, and had no correlation with patients’ age, T stage, N stage, tumor grade, and ER status in the validation cohort (Table 1). According to the TCGA breast cancer database, IGF2 expression level was significantly correlated with the HER2 status (Immunohistochemical type or gene PAM50 type). In addition, IGF2 expression level was also correlated with pathological stage, ER and PR status (Table 2). We also used immunohistochemical analysis method to show IGF2, p-IGF1R, p-Akt (S473) and PTEN expression in tumor tissues in the validation cohort (Figure 4B), the results revealed that the level of IGF2 and phosphoprotein were increased in the poor response group compared to good response group, but PTEN expression was just the opposite (Table 3). All the above results indicated that the higher IGF2 level was correlated with poor response to Herceptin in HER2 positive breast cancer. Further, we explored the relationship between miR-98-5p/IGF2 expression and prognosis of HER2-positive breast cancer patients by using Kaplan Meier plotter (https://kmplot.com/analysis/). The data showed that higher expression of IGF2 and/or lower expression of miR-98 were significantly correlated with poor overall survival (OS), recurrence free survival (RFS) and distant metastasis-free survival (DMFS) in HER2-positive breast cancer (Figure 4C).

**Table 1:** Association between IGF2 Expression and Clinicopathologic Characteristics in the Validation Cohort

characteristics	No.	IGF2 expression		χ^2^	p
		Low (~ +)	High (++ ~)		
Age					
≥50	21	13	8	0.351	0.554
<50	19	10	9		
T stage					
T2	29	16	13		
T3	6	3	3	0.411	0.814
T4	5	2	3		
N stage					
N0	11	7	4		
N1	23	11	12	0.764	0.683
N2	6	3	3		
Tumor grade					
G1	6	4	2		
G2	20	13	7	1.895	0.388
G3	14	6	8		
Therapeutic response					
CR+PR	25	18	7	4	0.046*
SD+PD	15	6	9		
HER2 status					
ICH 3+	22	13	9	0.852	0.356
ICH 2+ & FISH +	18	8	10		
ER & PR status					
+	16	9	7	0.017	0.896
-	24	14	10		

**Table 2:** IGF2 Expression Data from TCGA Database of BRCA in Different Clinicopathologic Characteristics

Characteristic	Low expression of IGF2	High expression of IGF2	p
T stage, n (%)			< 0.001
T1	124 (11.5%)	153 (14.2%)	
T2	347 (32.1%)	282 (26.1%)	
T3	46 (4.3%)	93 (8.6%)	
T4	21 (1.9%)	14 (1.3%)	
N stage, n (%)			0.133
N0	273 (25.7%)	241 (22.7%)	
N1	170 (16%)	188 (17.7%)	
N2	56 (5.3%)	60 (5.6%)	
N3	31 (2.9%)	45 (4.2%)	
M stage, n (%)			0.51
M0	451 (48.9%)	451 (48.9%)	
M1	12 (1.3%)	8 (0.9%)	
Pathologic stage, n (%)			0.008
Stage I	81 (7.6%)	100 (9.4%)	
Stage II	333 (31.4%)	286 (27%)	
Stage III	103 (9.7%)	139 (13.1%)	
Stage IV	11 (1%)	7 (0.7%)	
Age, n (%)			0.093
<=60	286 (26.4%)	315 (29.1%)	
>60	255 (23.5%)	227 (21%)	
HER2 status, n (%)			0.016
Negative	252 (34.7%)	306 (42.1%)	
Indeterminate	5 (0.7%)	7 (1%)	
Positive	91 (12.5%)	66 (9.1%)	
ER status, n (%)			< 0.001
Negative	165 (15.9%)	75 (7.2%)	
Indeterminate	2 (0.2%)	0 (0%)	
Positive	342 (33%)	451 (43.6%)	
PR status, n (%)			< 0.001
Negative	226 (21.9%)	116 (11.2%)	
Indeterminate	2 (0.2%)	2 (0.2%)	
Positive	281 (27.2%)	407 (39.4%)	
Histological type, n (%)			< 0.001
Infiltrating Ductal Carcinoma	438 (44.8%)	334 (34.2%)	
Infiltrating Lobular Carcinoma	45 (4.6%)	160 (16.4%)	
PAM50, n (%)			< 0.001
Normal	6 (0.6%)	34 (3.1%)	
LumA	178 (16.4%)	384 (35.5%)	
LumB	142 (13.1%)	62 (5.7%)	
Her2	63 (5.8%)	19 (1.8%)	
Basal	152 (14%)	43 (4%)	
Menopause status, n (%)			0.339
Pre	107 (11%)	122 (12.6%)	
Peri	17 (1.7%)	23 (2.4%)	
Post	359 (36.9%)	344 (35.4%)	

**Table 3:** Differences Proteins Expression between Two Groups in Tumor Tissue According to the Treatment Response

	Good response (n = 25)		Poor response (n = 15)		χ^2^	p
	High (++~+++)	Low (0~++)	High (++~+++)	Low (0~++)		
PTEN	19	6	6	9	5.184	0.023
p-IGF1R	5	20	9	6	6.593	0.01
p-Akt(S473)	6	19	10	5	7.111	0.008
IGF2	7	18	9	6	4	0.046

## Discussion

The importance of IGF-1R activation in the development of Herceptin resistance has been well documented (Jia et al., 2013; Lyu et al., 2016; Christodoulou et al., 2018; Lenz et al., 2018), but the exact mechanism leading to signaling pathway disorder or abnormal activation is still unclear. Searching for specific targets to reverse Herceptin resistance has seldom been reported. Human epidermal growth factor receptor 2 (HER2) is one of the HER family member. It lacks natural ligand in vivo unlike the other members (Oude Munnink et al., 2009). The formation of homologous with HER2 itself or heterodimer with other growth factor receptor like HER3 or IGF1R plays an important role to maintain the malignant phenotype, like invasion and proliferation (Murad et al., 2021; Yuan et al., 2021). Studies have found that IGF2/IGF-1R/IRS1 signal pathway is normally inhibited through negative feedback to maintain basic cell survival and proliferation in HER2-positive human breast cancer cells (Luo et al., 2021). In this study, we found that the negative feedback inhibition was disrupted due to significantly increased IGF-1R/HER2 heterodimer formation which directly led to Herceptin resistance in SKBR3-R cells.

Our results provide a new viewpoint to better understanding the molecular mechanisms of IGF2 mediated signaling pathway activation in Herceptin resistant breast cancer. Firstly, IGF2 overexpression could be seen in Herceptin resistant breast cancer cells. Second, a large number of IGF2 molecules bind to its ligand IGF-1R, triggering the activation of IGF-1R phosphorylation and leading to its configuration change. Finally, the activated IGF1R interacts with HER2 to form heterodimer on the surface of cell membrane. Although HER2 heterodimer formation is often reported, the specific mechanism remains to be further explored (Banappagari et al., 2012; Yu et al., 2017; Zhao et al., 2021). Insulin-like receptor substrate 1 (IRS1) is a specific substrate of IGF1R, so if IRS1 is phosphorylated we regard as the signal comes from IGF1R activation rather than HER2 (Farabaugh et al., 2016; Qiu et al., 2019; Misiura and Miltyk, 2020). Researchers have found that IGF-1R/IRS1 signaling pathway is also related to the adhesion process among malignant tumor cells, and plays an important role in the phenotype maintenance of cancer cells and the process of anti-tumor treatment resistance (Marconett et al., 2012; Yang et al., 2018; Luo et al., 2021). Our results were consistent with those of other studies. Therefore, we speculated that one of the mechanisms leading to SKBR3 cell resistant to Herceptin might begin with the increased level of IGF2, which then promoted the formation of IGF1-R/HER2 heterodimer and activated AKT/mTOR signaling pathway through the critical protein IRS1.

Interestingly, the IGF2 mRNA levels were almost the same, although IGF2 protein expression levels were significantly different in SKBR3-R cells and SKBR3 cells. This phenomenon indicated that IGF2 expression might be regulated during mRNA translation. MicroRNAs are known to be one of the most common non-coding RNAs in living organisms, mainly inhibits the translational activity of its target mRNA. In order to find specific microRNA that regulate IGF2 expression, we downloaded 3′ UTR sequences of IGF2 genes and miRNA family information from the Target Scan database http://www.targetscan.org/vert_72/. For each putative miRNA-UTR target site, Target Scan calculates a context++ score which takes into account both evolutionary conservation and targeting efficiency. We took the top weighted context score for each unique miRNA-UTR target pair. Among the candidate miRNAs, we are particularly interested in miR-98-5p. Meanwhile, miR-98-5p has been reported as a tumor suppressor gene to inhibit tumor growth and metastasis in a variety of cancers (Wu et al., 2019; Sun et al., 2020; Shi et al., 2021; Zhan et al., 2021), and associated with resistance to anti-tumor drug therapy (Wang et al., 2018). However, the relationship between miR-98-5p expression and Herceptin sensitivity has not been reported. In our research, we found the interreaction between miR-98-5p and IGF2 by luciferase reporter gene test, indicating that miR-98-5p is involved in the regulation of IGF2 expression. One of the important malignant phenotypes of tumor is invasion. MiR-98-5p can affect the invasiveness and drug sensitivity of cells by regulating the expression of IGF2. This is a very important discovery and provides a new idea for clinical treatment strategy to overcome Herceptin resistance. 

In vitro study results further led us to wonder whether such a mechanism exists in clinical patients with HER2-positive breast cancer who are developed resistance to Herceptin. We used TCGA databases and found that the expression level of IGF2 in breast cancer tissues was lower than that in normal tissues and had no correlation with the overall survival of breast cancer, the expression of IGF2 in HER2-positive breast cancer tissues was lower than that in HER2-negative breast cancer tissues, all the above suggesting that IGF2 may not be directly related to the occurrence and development of breast cancer. It may not be an independent biomarker. However interestingly, it seems that HER2-positive subtype was the only subgroup that had a prognostic association with IGF2 expression among the 4 molecular subgroups of breast cancer based on the TCGA database (including overall survival (OS), recurrence free survival (RFS) and distant metastasis-free survival (DMFS)). This suggests that IGF2 plays an important role in the development of HER2-positive breast cancer.

In the validation cohort, we found that serum IGF2 levels were significantly higher in poor response group than that in good response group among HER2 positive breast cancer patients, which were consistent with the results of postoperative immunohistochemistry report in tumor tissues. The increase of IGF2 protein seems to be directly related to the AKT/mTOR signaling pathway abnormal activation in patients with poor response. We know that patients with Herceptin resistance generally have a worse prognosis. Previous research from Denmark (Kalledsøe L et al., 2019 ) has shown that elevated serum IGF2 levels were not associated with short-term prognosis but a worse long-term prognosis after 5 to 10 years especially in certain subtypes like HER2 positive. Moreover, miR-98-5p might also be an important prognostic indicator for HER2-positive breast cancer, because the survival curve plotted by Kaplan Meier also showed that when miR-98-5p decreased the OS of patients was poor. Such results are also consistent with the internal relationship between miR-98-5p and IGF2.

In conclusion, our study investigated the biological role of miR-98-5p and IGF2 in HER2 positive breast cancer. MiR-98-5p is able to regulate the expression of IGF2 as a tumor suppressor gene, and ultimately affect the cell’s sensitivity to Herceptin. The transcriptional regulatory mechanisms affecting miRNA expression are unclear, further research is still needed. In the future, researchers may be able to design a particular kind of agent targeting miR-98-5p to overcome drug resistance during Herceptin therapy.

## Author Contribution Statement

Study concept and design: Zhang Mingling. Acquisition of data: Li Zhejiang, Liu Xianzu. Statistical analysis: Liu Xianzu. Drafting of the manuscript: Zhang Mingling, Li Zhejiang, Liu Xianzu. Study supervision: Zhang Mingling. 

## Statement conflict of Interest

The authors declare that there is no conflict of interest. 
